# RETRACTION: In Vitro Efficacy of Rosa Damascena Solid State Fermentation Liquid and Water Extract on Skin Care

**DOI:** 10.1111/srt.70217

**Published:** 2025-08-25

Chen Z, Hong N, Xu N, Yan C, Cao P, Yao H. In vitro efficacy of Rosa damascena solid state fermentation liquid and water extract on skin care. *Skin Res Technol*. 2024;30(8):e13869. doi:10.1111/srt.13869

The above article, published online on 22 August 2024 in Wiley Online Library (wileyonlinelibrary.com), has been retracted by agreement between *Skin Research and Technology*; and John Wiley & Sons Ltd. The article was accepted for publication following an insufficient peer review process that does not meet the standards of the journal's peer review policy. Furthermore, scientific inconsistencies between methodology described and results presented were found. As a result, the editorial team cannot vouch for the reliability of the study's conclusions. Accordingly, the article has been retracted. The authors have been informed of this decision but unavailable for a final confirmation.

